# Investigation of Material Constants of CaTiO_3_ Doped (K,Na)NbO_3_ Film by MEMS-Based Test Elements

**DOI:** 10.3390/mi9110558

**Published:** 2018-10-29

**Authors:** Ryosuke Kaneko, Michio Kadota, Yuji Ohashi, Jun-ichi Kushibiki, Shinsuke Ikeuchi, Shuji Tanaka

**Affiliations:** 1 Graduate School of Engineering, Tohoku University, 6-6 Aramaki aza, Aoba, Aoba-ku, Sendai, Miyagi 980-8579, Japan; mkadota@mems.mech.tohoku.ac.jp (M.K.); tanaka@mems.mech.tohoku.ac.jp (S.T.); 2 New Industry Creation Hatchery Center, Tohoku University, 6-6 Aramaki aza, Aoba, Aoba-ku, Sendai, Miyagi 980-8579, Japan; ohashi@imr.tohoku.ac.jp; 3 Graduate School of Electrical Engineering, Tohoku University, 6-6 Aramaki aza, Aoba, Aoba-ku, Sendai, Miyagi 980-8579, Japan; kushi@ecei.tohoku.ac.jp; 4 Murata Manufacturing Co., Ltd., 1-10-1 Higashikotari, Nagaokakyo, Kyoto 617-8555, Japan; s_ikeuchi@murata.com

**Keywords:** KNN-CT film, material constants, piezoelectric resonance, leaky Lamb wave, LFB-UMC, genetic algorithm

## Abstract

A CaTiO_3_-doped (K,Na)NbO_3_ (KNN-CT) film is a lead-free piezoelectric film that is expected to substitute Pb(Zr,Ti)O_3_ (PZT) film in piezoelectric micro electro mechanical systems (MEMS). However, the full set of the material constants (elastic constants, piezoelectric constants and dielectric constants) of the KNN-CT film have not been reported yet. In this study, all the material constants of a sputter-deposited blanket KNN-CT film were investigated by the resonance responses of MEMS-based piezoelectric resonators and the phase velocities of leaky Lamb waves on a self-suspended membrane. The phase velocities measured by a line-focus-beam ultrasonic material characterization (LFB-UMC) system at different frequencies were fitted with theoretical ones, which were calculated from the material constants, including fitting parameters. A genetic algorithm was used to find the best-fitting parameters. All the material constants were then calculated. Although some problems arising from the film quality and the nature of deliquescence are observed, all the material constants were obtained exhibiting accuracy within 16 m/s in the phase velocity of leaky Lamb wave.

## 1. Introduction

Pb(Zr,Ti)O_3_ (PZT) films are used for commercialized piezoelectric micro electro mechanical systems (MEMS), such as ink-jet printer heads and vibratory gyroscopes, due to their well-balanced performance. However, PZT includes lead (Pb) at high concentration, which may pollute the environment and eventually harm the human body. At present, there is no good replacement of PZT from a practical point of view. Therefore, lead-free piezoelectric films are important targets of research.

(K,Na)NbO_3_ (KNN) is a replacement candidate for PZT [1,2,3]. Shibata et al. investigated KNN films deposited by RF magnetron sputtering [4,5,6,7,8,9]. The sputtered KNN film on Pt/Ti/SiO_2_/Si exhibited excellent transverse piezoelectric coefficient *e*_31_* from −10.0 to −14.4 C/m^2^ compared to other lead-free piezoelectric materials [8]. Besides pure KNN, different kinds of KNN-based materials have also been widely studied [10,11]. Ikeuchi et al. reported that a CaTiO_3_-doped KNN (KNN-CT) film deposited by RF sputtering showed excellent piezoelectric characteristics (*e*_31_* = −11.7 C/m^2^) [12]. This level of performance can open the application of the KNN-CT film to piezoelectric MEMS. However, material constants (elastic, piezoelectric and dielectric) of the KNN-CT film, which are required for simulation to design MEMS, have not been revealed. For ceramic and single crystal KNN, full material constants have been reported [13,14]. The full material constants of a piezoelectric film, however, are difficult to determine, and no one has reported them for a KNN-CT film.

**Figure 1:**
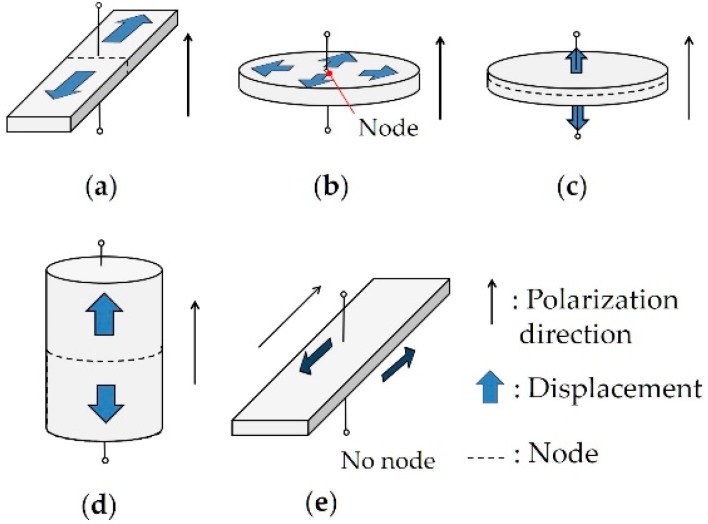
Piezoelectric resonance modes relative to polarization direction and displacement. (**a**) Length expander mode. (**b**) Radial expander mode. (**c**) Thickness longitudinal mode. (**d**) Cylinder longitudinal mode. (**e**) Thickness shear mode.

The methods to determine all the material constants of a piezoelectric films have been proposed. Konno et al. reported full material constants of a ScAlN film [15], which were determined using MEMS-based bulk acoustic resonators and a line-focus-beam ultrasonic material characterization (LFB-UMC) system [16,17]. This method is partly based on that for bulk ceramics [18]. Figure 1 shows each resonance mode relative to the polarization direction. Some of the material constants are measured by a length expander mode resonator (Figure 1a), a radial expander mode resonator (Figure 1b) and a thickness longitudinal mode resonator (Figure 1c). However, all the material constants of a film cannot be determined as those of ceramics, because it is impossible to fabricate a cylinder longitudinal mode resonator (Figure 1d) or a thickness shear mode resonator (Figure 1e) using the c-axis oriented film. Therefore, the phase velocity of the leaky Lamb wave on a self-suspended film was measured using the LFB-UMC system, and parameter fitting using a theoretical model was then done to determine the material constants that were not directly measured [15].

Parsapour et al. determined *c*_33_ and *c*_44_ of ScAlN, which were derived from the cylinder longitudinal mode and thickness shear mode, by using a dual mode bulk acoustic wave resonator with a tilted c-axis oriented film [19]. The tilted c-axis oriented ScAlN film was grown on a non-electrode, i.e., insulating layer. Both methods by Konno et al. and Parsapour et al., however, have a limitation in deposition; the bottom electrode layer must be patterned before the deposition of a piezoelectric film. These methods cannot be used for a blanket piezoelectric film on a fully metal-coated bottom layer, which is often better in terms of film quality.

In this study, we investigated the material constants of a blanket KNN-CT film on a 4-inch wafer using acoustic resonators and the LFB-UFC system, inspired by [15]. In a previous study, ScAlN was deposited on a Si substrate, where Pt bottom electrodes were patterned in advance. In this study, however, a blanket KNN-CT film deposited on a fully Pt-coated Si substrate without a pattern was characterized. This structure of sample is more general than the previous ones.

In addition, genetic algorithm was applied for the fitting of the phase velocity of the leaky Lamb wave. The fitting is an optimization technique to minimize errors between a theoretical model and measured data using several fitting parameters. For such an optimization problem, initial values should be close to global solutions to avoid the risk of local convergence. Compared to ScAlN, few material constants of KNN-CT were reported at present. Genetic approaches such as crossover, mutation and selection were introduced to escape local convergence and reach optimized fitting parameters from relatively far initial values.

## 2. Measurement Methods and Theoretical Equations

### 2.1. Piezoelectric Resonators

Piezoelectric resonators are used to measure the material constants for C_6v_ and C_∞v_ crystal structures, including c-axis oriented KNN. All material constants of bulk ceramics can be determined from series and parallel resonance frequencies (*f*_s_ and *f*_p_) of the following modes of resonators: length expander, radial expander, cylinder longitudinal, thickness longitudinal and thickness shear [18]. For the length expander mode, *f*_s_ and electromechanical coupling coefficient (coupling factor) *k*_31_ are expressed as:

(1) $$f_{s}=\frac{1}{2l\sqrt{\rho s_{11}^{E}}},$$

(2) $$\frac{k_{31}{}^{2}}{1-k_{31}{}^{2}}=-\frac{\pi }{2}\cdot \frac{f_{p}}{f_{s}}\cot\left(\frac{\pi }{2}\cdot \frac{f_{p}}{f_{s}}\right)\;\mathrm{and}$$

(3) $$k_{31}=\frac{d_{31}}{\sqrt{s_{11}^{E}\varepsilon_{33}^{T}}},$$

where *l* and *ρ* are the length of the resonator and density, respectively. *s*_11_^E^, *d*_31_, and *ε*_33_^T^ are elastic compliance at electric field *E* = 0, piezoelectric *d* constant and dielectric constant in stress-free condition (*T* = 0), respectively.

For the radial expander mode, *f*_s_ and coupling factor *k*_r_ are expressed as:

(4) $$f_{s}=\frac{\phi_{1}}{2\pi r}\cdot \frac{1}{\sqrt{\rho s_{11}^{E}\left(1-\sigma^{E^{2}}\right)}}\;\mathrm{and}$$

(5) $$k_{r}=d_{31}\sqrt{\frac{2}{\left(s_{11}^{E}+s_{12}^{E}\right)\varepsilon_{33}^{T}}}=k_{31}\sqrt{\frac{2}{1-\sigma^{E}}},$$

where *r* and *σ*^E^ are the radius of the resonator and Poisson’s ratio, respectively. *s*_12_^E^ is elastic compliance at *E* = 0. $\phi_{1}$ is a constant determined by *σ*^E^. Elastic compliance *s*_66_^E^ is calculated from *s*_11_^E^ and *s*_12_^E^ as *s*_66_^E^ *=* 2(*s*_11_^E^ − *s*_12_^E^).

For the thickness longitudinal mode, *f*_p_ and coupling factor *k*_t_ are expressed as:

(6) $$f_{p}=\frac{1}{2h}\sqrt{\frac{c_{33}^{D}}{\rho }},$$

(7) $$k_{t}{}^{2}=\frac{\pi }{2}\cdot \frac{f_{s}}{f_{p}}\cot\left(\frac{\pi }{2}\cdot \frac{f_{s}}{f_{p}}\right)\;\mathrm{and}$$

(8) $$c_{33}^{E}=\frac{c_{33}^{D}}{1-k_{t}{}^{2}},$$

where *h*, *c*_33_^D^ and *c*_33_^E^ are the thickness of the resonator and elastic stiffness constants at electrical displacement *D* = 0 and *E* = 0, respectively.

By measuring free capacitance *C*_f_ of the radial expander mode resonators, the relative dielectric constant at *T* = 0, *ε*_33_^T^⁄*ε*_0_, is calculated as:

(9) $$\frac{\varepsilon_{33}^{T}}{\varepsilon_{0}}=\frac{C_{f}h}{\varepsilon_{0}\pi r^{2}},$$

where *r* and *ε*_0_ are the radius of the resonator and the dielectric constant of vacuum, respectively.

Using the above resonators, *s*_11_^E^, *s*_12_^E^, *s*_66_^E^, *c*_33_^E^, *k*_31_, *k*_r_, *k*_t_, *d*_31_, *σ*^E^ and *ε*_33_^T^ are determined. Elastic compliance *s*_33_^E^, coupling factor *k*_33_ and piezoelectric d constant *d*_33_ are obtained by the cylinder longitudinal resonator, and elastic stiffness *c*_44_^E^, coupling factor *k*_15_, elastic compliance *s*_44_^E^, dielectric constant *ε*_11_^T^ and piezoelectric d constant *d*_15_ are obtained by the thickness shear resonator. All other constants (e.g., elastic compliance *s*_13_^E^, elastic stiffness *c*_11_^E^, *c*_12_^E^, *c*_13_^E^ and *c*_66_^E^, piezoelectric e constants *e*_31_, *e*_33_ and *e*_15_, dielectric constants in strain-free condition (*S* = 0) *ε*_11_^S^ and *ε*_33_^S^) are obtained from the above constants. The detailed flow is described in [18]. However, both cylinder longitudinal and thickness shear resonators cannot be fabricated using a c-axis oriented KNN-CT film. Instead, the phase velocity of leaky Lamb wave in a suspended film is used, as described in Section 3.3.

### 2.2. Phase Velocities of Leaky Lamb Wave

The phase velocity of leaky Lamb wave is affected by most material constants. The relationship between the phase velocity and the material constants is not explicit; however, the material constants not available from the resonators can be determined by fitting the calculated phase velocity to the measured one in terms of different modes of leaky Lamb wave [15].

**Figure 2:**
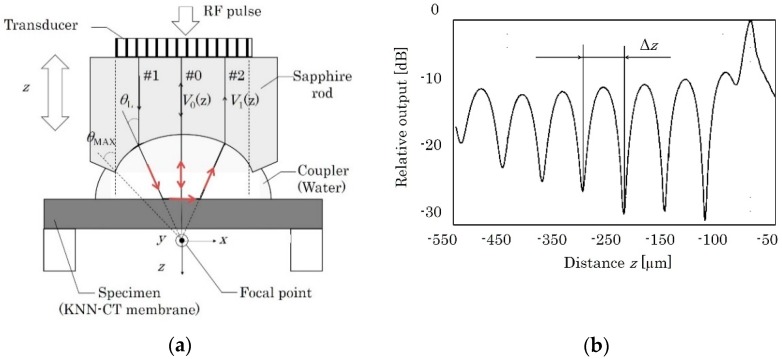
Schematic representation of the LFB-UMC system. (**a**) Cross-sectional geometry of the LFB acoustic lens and specimen to show *V*(*z*) curve measurement. (**b**) Typical *V*(*z*) curve as a transducer output.

The phase velocity of leaky Lamb wave is measured by the LFB-UMC system. Figure 2a shows the schematics of the LFB-UMC system. Longitudinal bulk wave is generated by applying RF pulse to the transducer, focused on by the cylindrical lens at the end of the sapphire rod. The focused beam is incident on the specimen, i.e., the self-suspended KNN-CT film, via the water coupler (Path #1), and then converted into leaky Lamb waves. The leaky Lamb waves propagate in the suspended film, emitting acoustic energy into water. The emitted bulk waves return to the transducer via Path #2. On the other hand, the normal incident wave reflects vertically at the specimen surface and returns via Path #0.

By changing the relative distance *z* between the sapphire rod lens and the specimen, the transducer output *V*(*z*) curve is obtained, as illustrated in Figure 2b. After the removal of background and lens characteristics and the extraction of an interference component from *V*(*z*) curve, the oscillation interval Δ*z* is obtained by fast Fourier transform (FFT). Finally, the phase velocity of LSAW *V*_L_ is given as:

(10) $$V_{L}=\frac{V_{w}}{\sqrt{1-{\left(1-\frac{V_{w}}{2f\Delta z}\right)}^{2}}},$$

where *V*_W_ and *f* are the longitudinal wave velocity of water and measurement frequency, respectively.

### 2.3. Theoretical Phase Velocity of Lamb Wave

**Figure 3:**
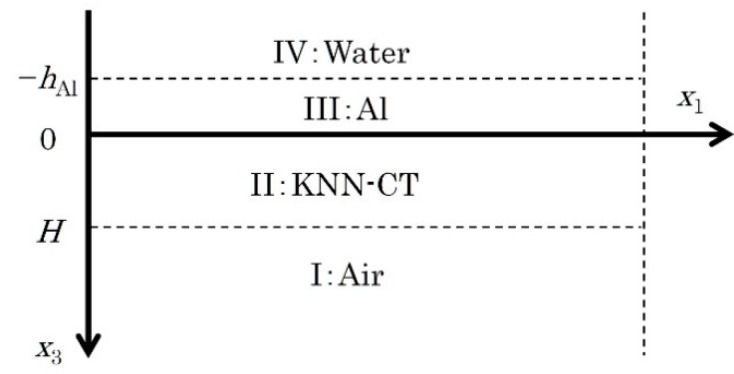
Analysis model of leaky Lamb wave propagation on self-suspended Al-metallized KNN-CT membrane. Lamb wave propagates to *x*_1_ direction.

The theoretical phase velocity of Lamb waves is calculated by Campbell’s method [20]. In the LFB-UMC system, a water coupler exists on one side of the membrane, as shown in Figure 2a, and thus Campbell’s method is extended to calculate the phase velocities of leaky Lamb wave. Figure 3 shows an analysis model used in this study. The top of the KNN-CT membrane is covered with Al, which is exposed to the water coupler. *H* and *h*_Al_ are thickness of the KNN-CT membrane and Al, respectively. Acoustic wave propagation in this model is governed by Hook’s law (Equation (11)) for non-piezoelectric materials (air, Al and water) or piezoelectric fundamental equations (Equations (12) and (13)) for KNN-CT, Maxwell’s equation (Equation (14)) and Newton’s equation of motion (Equation (15)).

(11) $$T_{i}=c_{ij}^{E}S_{j}$$

(12) $$T_{i}=c_{ij}^{E}S_{j}-e_{mi}E_{m}$$

(13) $$D_{n}=e_{nj}S_{j}+\varepsilon_{nm}^{T}E_{m}$$

(14) div D=0

(15) $$\rho \frac{\partial^{2}u_{h}}{\partial t^{2}}=\frac{\partial T_{hk}}{\partial x_{k}}$$

*T*, *S*, *E*, *D*, *u* and *t* are stress, strain, electric field, electric displacement, particle displacement and time, respectively. *x* is the propagation direction of acoustic wave. The suffixes (*i*, *j*), (*n*, *m*) and (*h*, *k*) take 1–6, 1–3 and 1–3, respectively. *S* and *E* are expressed as:

(16) $$S_{hk}=\frac{1}{2}\left(\frac{\partial u_{h}}{\partial x_{k}}+\frac{\partial u_{k}}{\partial x_{h}}\right)\;\mathrm{and}$$

(17) $$E_{m}=-\frac{\partial \varphi }{\partial x_{m}},$$

where *φ* is an electric potential. Note that *S_j_* is the abbreviated notation of *S_hk_*. The particle displacements *u*_1_ and *u*_3_, and *φ* of laterally propagating acoustic waves in each layer are assumed as follows:

(18) $$u_{1}^{\alpha }=A^{\alpha }\exp\left(\Omega^{\alpha }kx_{3}\right)\exp\left\{j\left(kx_{1}-\omega t\right)\right\}$$

(19) $$u_{3}^{\alpha }=C^{\alpha }\exp\left(\Omega^{\alpha }kx_{3}\right)\exp\left\{j\left(kx_{1}-\omega t\right)\right\}$$

(20) $$\varphi^{\alpha }=D^{\alpha }\exp\left(\lambda^{\alpha }kx_{3}\right)\exp\left\{j\left(kx_{1}-\omega t\right)\right\}$$

*A^α^*, *C^α^* and *D^α^* are amplitudes, Ω*^α^* and *λ^α^* are decay constants, and *k* and *ω* are wave number and angular frequency, respectively. The superscript *α* indicates each layer (I–IV) in Figure 3.

For non-piezoelectric film layers (I, III and IV), Equations (18) and (19) are substituted for the displacements in Newton’s equation of motion (Equation (15)) using Equations (11) and (16). The eigenvalues are obtained from the characteristic equation of Ω*^α^* as Ω*_l_*^I^ (*l* = 1–2), Ω*_m_*^III^ (*m* = 1–4) and Ω^IV^. Note that only negative Ω*_l_*^I^ and positive Ω^IV^ are adopted to prevent divergence at positive and negative infinity in *x*_3_ direction, respectively. By substituting Equation (20) for the electric potential in Maxwell’s equation (Equation (14)) using Equation (17), the eigenvalues are obtained from the characteristic equation of *λ^α^* as *λ*^I^, *λ_l_*^III^ and *λ*^IV^. Note that only negative *λ*^I^ and positive *λ*^IV^ are adopted for the same reason. For the piezoelectric film layer (II), Equations (18)–(20) are substituted for the displacements and electric potential in Equations (14) and (15) using Equations (12), (13), (16) and (17). In a similar way, eigenvalues of Ω*_n_*^II^ (*n* = 1–6) and *λ_l_*^II^ are obtained.

The general solutions of Equations (14) and (15) are given by Equations (18)–(20) with the eigenvalues for each layer. The boundary conditions summarized in Table 1 are applied to the general solutions, and a simultaneous equation in terms of *A_l_*^I^, *A_n_*^II^, *A_m_*^III^ and *A*^IV^ are obtained. This equation has non-zero, i.e., nontrivial, solutions at particular *v* = *ω*/*k*, where *v* is a complex phase velocity with an imaginary part of leaky component. *v* is numerically searched assuming the material constants of the KNN-CT, Al and water. By repeating this calculation at different *ω*, the frequency characteristic of the phase velocity is obtained.

### 2.4. Parameter Fitting Using Genetic Algorithm

All of the material constants are determined by solving the optimization problem to minimize the error between the measured phase velocities of leaky Lamb wave and the theoretical model. The parameters to be fitted are related to the cylinder longitudinal mode and the thickness shear mode, which are not measured by the piezoelectric resonators. Unfortunately, proper initial values are not available in this study because a few material constants of KNN-CT have been reported. Least squares methods (Newton method, Gauss-Newton method or Levenberg-Marquardt method) are not options to prevent local convergence. 

In this study, genetic algorithm was employed for parameter fitting. Genetic algorithm explores the best solutions of such optimization problems based on the mechanisms of natural selection, genetic combining, and mutation [21,22]. The role of mutation is to escape local convergence. Here, let fitting parameters be specific material constants, *k*_33_, *k*_15_, *s*_13_^E^, *c*_44_^E^ and *d*_15_. A solution vector of integer number and that of binary number to operate genetic algorithm are called *individual* and *chromosome*, respectively. The chromosomes are made of discrete units, called *genes*. *Population* is used as a collection of the individuals or the chromosomes.

**Figure 4:**
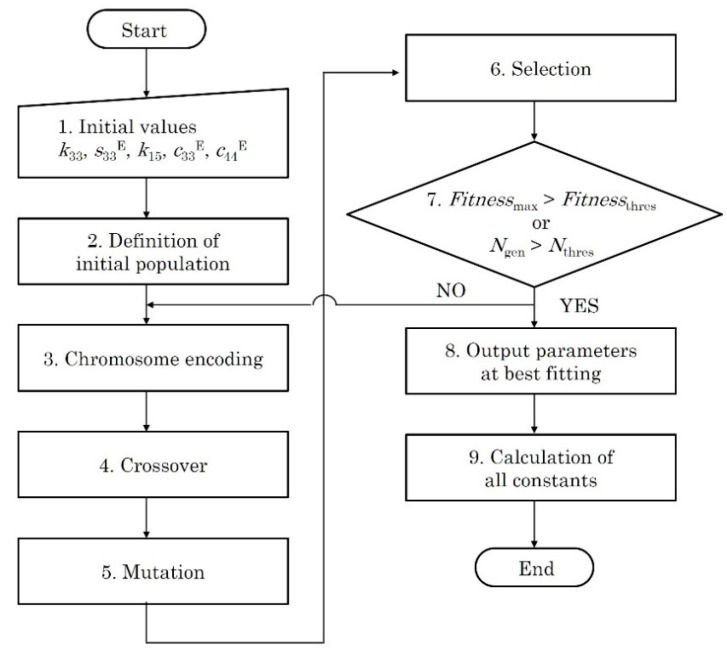
Flow chart of genetic algorithm for parameter fitting.

Figure 4 shows the flow chart of the developed algorithm using genetic algorithm. The algorithm starts with setting the initial values of the fitting parameters (step 1). Initial population with size *M* is then defined for each fitting parameter (step 2). The initial value of each fitting parameter is transformed into 3-digits integer part and index part. The integer part is used for individuals in the population, whereas the index part is used later to transform individuals into the fitting parameters. Random integer matrix (*M* × 1), which is 100 times multiplied Gauss-Laplace random number (mean 0, variance 1), is defined, and then each row of the matrix is added to the integer part to form the initial population. The initial population of each fitting parameter is formed independently in this way. Next, the individuals in the initial populations are transformed into binary digits to express as chromosomes (chromosome encoding, step 3). The populations of binary chromosomes are defined in this way.

Next, two of the chromosomes are selected randomly for crossover among the respective populations (step 4). The selected chromosomes become *parents* to form new *chromosomes* (*offspring*) with specific crossover probability *P*_c_. The selection is repeated until the number of pair of parents becomes *M*. As a crossover method, uniform crossover is employed, which forms the chromosomes of offspring by exchanging each gene in a chromosome of parents at specific probability *P*_u_ (50%). The size of the population for offspring becomes 2*M* × 1 since two offspring are formed from single pair of parents. The crossover is conducted on each population independently. After that, the genes of chromosomes among the offspring are randomly changed at specific probability *P*_m_ as mutation, which makes it possible to escape local convergence (step 5).

In step 6, natural selection is done by selecting chromosomes with the number *M* among the offsprings toward a parameter, which is called *fitness*. Fitness is defined with theoretical phase velocities, thus the fitting parameters of the offspring are necessary. To obtain fitting parameters, the individuals of the offspring are calculated by transforming the binary chromosomes into a decimal integer. The fitting parameters of the offsprings are then calculated by multiplying the individuals with the index part, which was removed at the definition of initial populations. The populations of fitting parameters of offspring are defined in this way. After that, the combinations of fitting parameters (e.g., *k*_33_, *k*_15_, *s*_13_^E^, *c*_44_^E^ and *d*_15_), which are the candidates of the solutions in this generation, are formed in the same row of the populations. The theoretical phase velocities are then calculated for each combination, according to the calculation described in Section 2.3.

Fitness is defined as an inverse function of the squared error between the calculated frequency characteristic and measured one, as Equation (21).

(21) $$\mathrm{Fitness}=\frac{1}{{\left(v_{A_{0},\;\mathrm{meas}}-v_{A_{0},\;\mathrm{cal}}\right)}^{2}+{\left(v_{S_{0},\;\mathrm{meas}}-v_{S_{0},\;\mathrm{cal}}\right)}^{2}},$$

where the suffixes “meas”, “cal”, A_0_ and S_0_ represent measured value, calculated value, antisymmetric (A_0_) mode, and symmetric (S_0_) mode, respectively. Tournament selection was employed to select an offspring with the number *M*, in which combinations of fitting parameters with a specific number (tournament size) are randomly selected; a combination with the best fitness was selected as a winner [23,24]. The selection is repeated until the winning number, which is also called the next generation, becomes *M*. Finally, maximum fitness *Fitness*_max_ in the next generation is compared with threshold *Fitness*_thres_ (step 7). If the maximum fitness is less than the threshold, the populations of the next generation return to the chromosome encoding for the next calculation. Else, the fitting parameters of the best fitness are output as the best solution (step 8) and then substituted to calculate all material constants (step 9) [18]. The loop is also broken if the number of generation *N*_gen_ become a specific number *N*_thres_.

## 3. KNN-CT Film Deposition and Device Fabrication

### 3.1. KNN-CT Film Deposition

KNN-CT was deposited by RF sputtering on a 4 inch Si wafer of 500 µm thickness. As a seed layer, 150 nm thick SiO_2_, 10 nm thick Ti and 100 nm thick Pt were deposited in advance. The sputtering was done at a substrate temperature of 550 °C and a chamber pressure of 0.3 Pa using Ar and O_2_ with an O_2_/(Ar + O_2_) ratio of 0.01. The substrate temperature was optimized with film stress and crystallinity. Post annealing followed at 700 °C in O_2_ atmosphere for 10 min. The as-deposited film was self-polarized, as is often the case with sputtered piezoelectric films.

**Figure 5:**
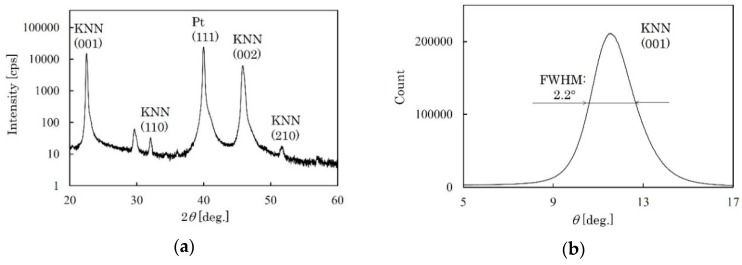
XRD results of KNN-CT film. (**a**) XRD pattern. (**b**) Rocking curve.

**Figure 6:**
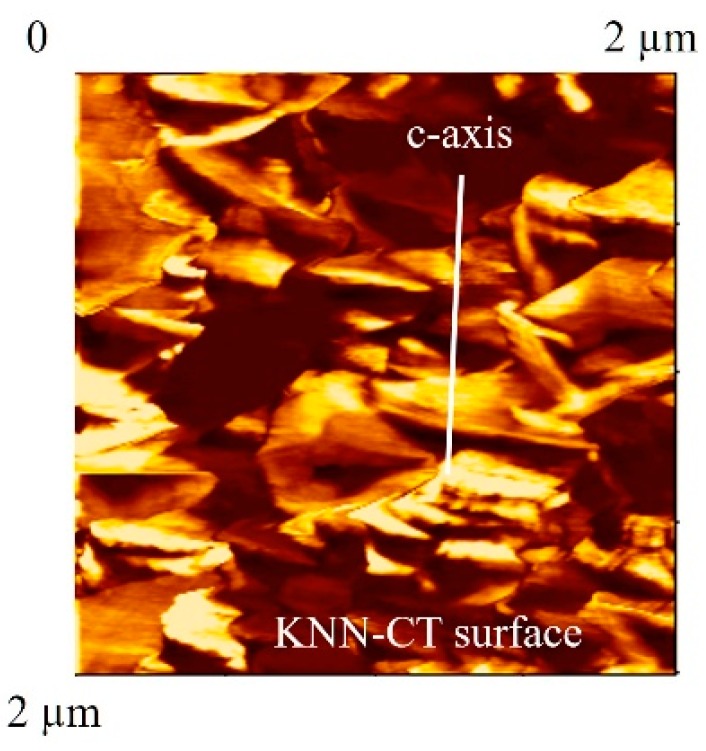
Polarity distribution of KNN-CT film. Bright and dark areas show c-axis orientation and other orientation, respectively.

The crystal orientation of the KNN-CT film was analyzed by X-ray diffraction (XRD). Figure 5a,b show the XRD pattern and rocking curve, respectively. Although the main peak is (001), (110) and (210) peaks are also found. The full-width of half-maximum (FWHM) of (001) peak is 2.2°. This result suggests that the KNN-CT film is partially c-axis oriented. In addition, scanning nonlinear dielectric microscopy (SNDM) was conducted to analyze the distribution of polarity. The detail of SNDM is described in [25]. Figure 6 shows the image of SNDM, where the blight and dark areas show c-domain and other domains, respectively. c-domain occupies 50–60% of the area.

The density of the KNN-CT film was calculated from the difference of the mass and volume of the 4-inch wafer, before and after KNN-CT deposition, measured by the Archimedes method [26]. As a result, the density and thickness of the KNN-CT film were obtained as 4.46 × 10^3^ kg/m^3^ and 1.75 µm, respectively.

### 3.2. Piezoelectric Resonators

The resonators of length expander mode, radial expander mode and thickness longitudinal mode were fabricated using MEMS process. The radial expander mode and thickness longitudinal mode were excited on the same resonator at different frequencies. The Pt seed layer was utilized as the bottom electrode of the resonators.

**Figure 7:**
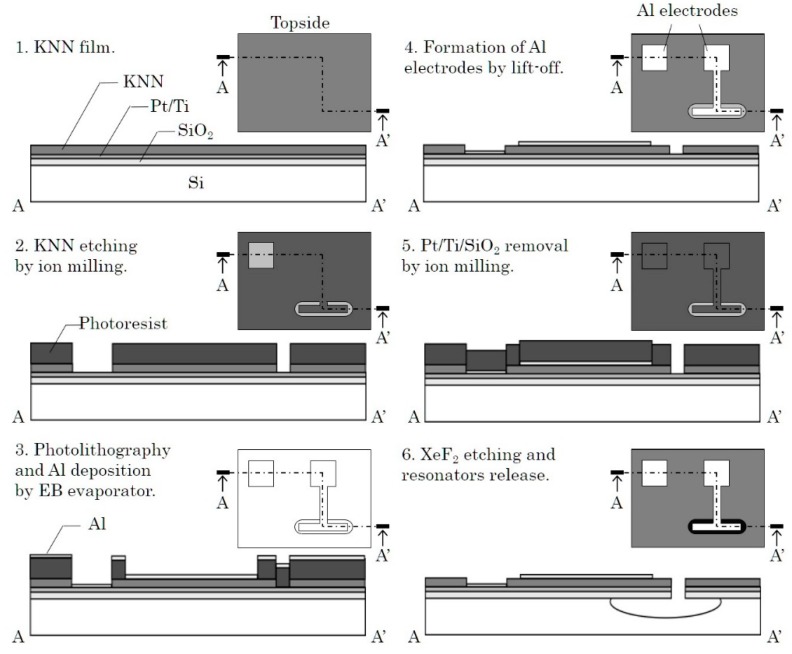
Process flow of piezoelectric resonators.

**Figure 8:**
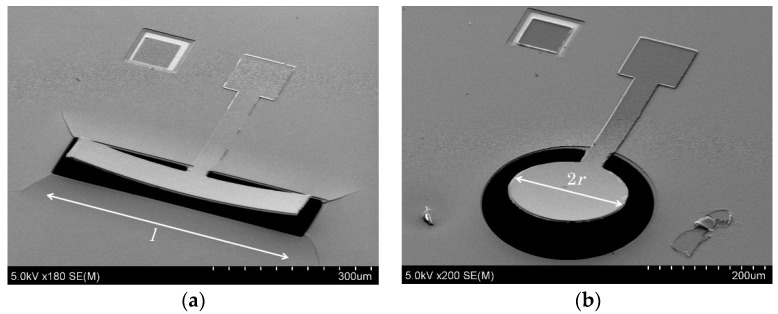
Piezoelectric resonators. (**a**) Length expander mode, *l* = 200–600 µm. (**b**) Radial expander mode and thickness longitudinal mode, *r* = 15–100 µm.

Figure 7 summarizes the fabrication process of the piezoelectric resonators. First, the KNN-CT-coated wafer was diced into 20 × 20 mm^2^ chips (1 of Figure 7). The KNN-CT film was etched by ion milling using a photoresist mask (2). Al top electrodes and the contacts of the bottom electrode of 200 nm thickness were formed by EB evaporation and lift-off process (3 and 4). Next, Pt/Ti/SiO_2_ layers were removed by ion milling to open etching windows for the following step (5). XeF_2_ etching was used to release the resonators at the final step (6). Figure 8a,b show the piezoelectric resonators of the length expander mode and the radial expander/thickness longitudinal mode, respectively. The distortion of the resonators happened after the release step, due to the film stress of the KNN-CT film.

### 3.3. Leaky Lamb Wave Device

**Figure 9:**
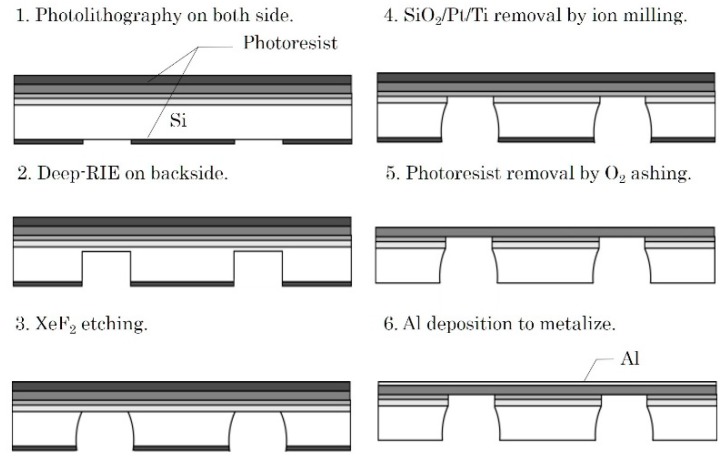
Process flow of self-suspended Al-metallized KNN-CT membrane for leaky Lamb wave.

**Figure 10:**
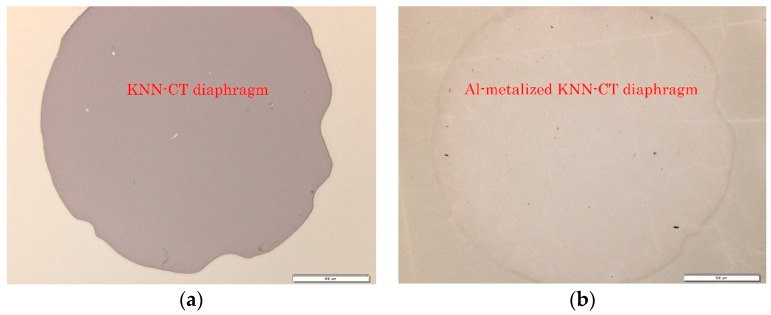
Topside microscopy images of KNN-CT membrane. The diameter is designed as 2 mm. (**a**) After O_2_ ashing. (**b**) After Al deposition.

A self-suspended Al-metalized KNN-CT membrane with a diameter of 2 mm was fabricated as a specimen for the LFB-UMC measurement. Figure 9 shows the fabrication process. First, the topside of the Si substrate, i.e., the KNN-CT film, was covered with photoresist. After photolithography on the backside, the Si substrate was etched by deep reactive ion etching (RIE) (1 and 2 of Figure 9). The etching was stopped before it reached the SiO_2_ layer to avoid damaging KNN-CT. Si left on the bottom of the etched hole was removed by XeF_2_ etching, and then the SiO_2_/Ti/Pt layers were removed by ion milling (3) and (4). The photoresist on both sides was removed by O_2_ ashing (5). Figure 10a shows microscope images of the KNN-CT membrane after O_2_ ashing. Finally, Al was deposited on the topside by EB evaporation (6), as shown in Figure 10b. The Al layer works as protection of the KNN-CT film from water absorption.

## 4. Results

### 4.1. Measurement of Piezoelectric Resonators

**Figure 11:**
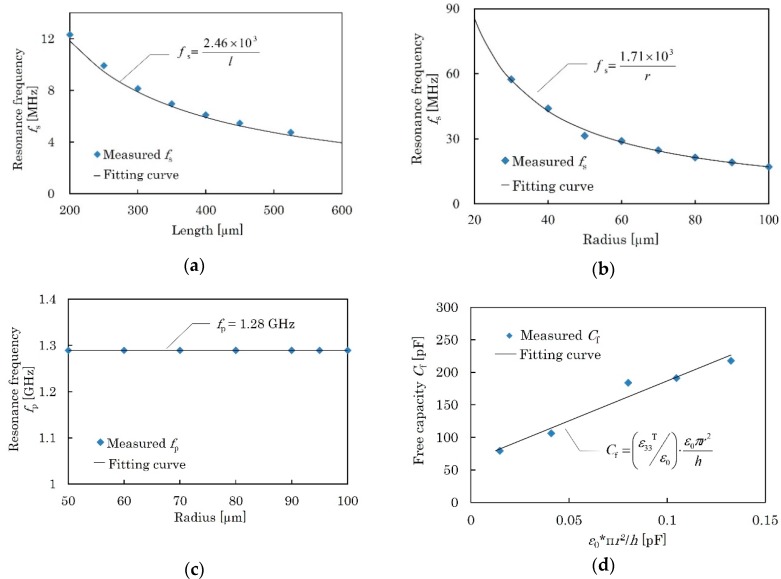
Measurement results of piezoelectric resonators. (**a**) Relationship between *f*_s_ and length of length expander mode resonator. (**b**) Relationship between *f*_s_ and radius of radial expander mode resonator. (**c**) Relationship between *f*_p_ and radius of thickness longitudinal mode resonator. (**d**) Relationship between free capacity *C*_f_ and radial function of radial expander mode resonator. *ε*_33_^T^/*ε*_0_ is obtained from the tilt of the fitting curve.

*f*_s_ and *f*_p_ of the piezoelectric resonators were measured by a network analyzer (E5071B, Agilent, Santa Clara, CA, USA). Figure 11a shows the relationship between the resonator length (*l* in Figure 8a) and *f*_s_ for the length expander mode. As predicted by theory, *f*_s_ is in inverse proportion to the length. From the best fit of Equation (1), *s*_11_^E^ = 9.27 pm^2^/N is obtained, and *k*_31_^2^ = 0.06% is obtained from Equation (2).

Figure 11b shows the relationship between the resonator radius (*r* in Figure 8b) and *f*_s_ for the radial expander mode. *f*_s_ is in inverse proportion to the radius. From the best fit of Equation (4), *σ*^E^ = 0.33, *s*_12_^E^ = −*σ*^E^*s*_11_^E^ = −3.06 pm^2^/N and *s*_66_^E^ = 24.7 pm^2^/N are obtained, and *k*_r_^2^ = 0.72% is obtained from Equation (5).

Figure 11c shows the relationship between the resonator radius (*r* in Figure 8b) and *f*_p_ for the thickness longitudinal mode. The resonator response is very small, probably due to poor orientation of the KNN-CT film. *f*_p_ is independent of the radius, showing that the measured response is for the thickness longitudinal mode. *c*_33_^D^ = 89.8 GPa and *k*_t_^2^ = 2.08% are obtained from Equations (6) and (7), respectively, and *c*_33_^E^ = 89.8 GPa is obtained from Equation (8).

Figure 11d shows the free capacitance *C*_f_ of the same type of resonators in terms of *ε*_0_π*r*^2^/*h*. As found in Equation (9), the relationship should be linear, and *ε*_33_^T^/*ε*_0_ = 1.27 × 10^3^ is obtained by the least-squares method. Accordingly, *d*_31_ = −78.9 pC/N is calculated from Equation (3).

### 4.2. Phase Velocity Measurement by the LFB-UMC System

**Figure 12:**
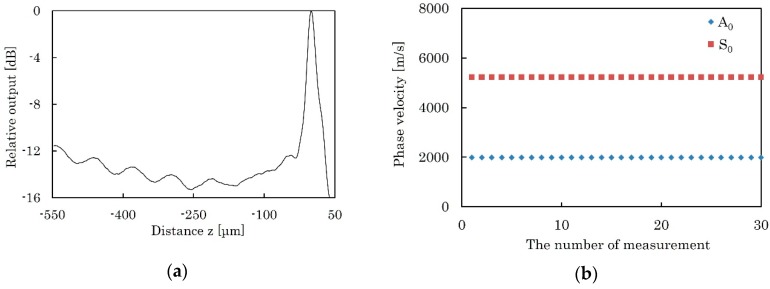
Measurement results by the LFB-UMC system. (**a**) Typical *V*(*z*) curve measured for the KNN-CT film at 225 MHz. (**b**) Transition of phase velocity of A_0_ and S_0_ modes, measured 30 times.

**Figure 13:**
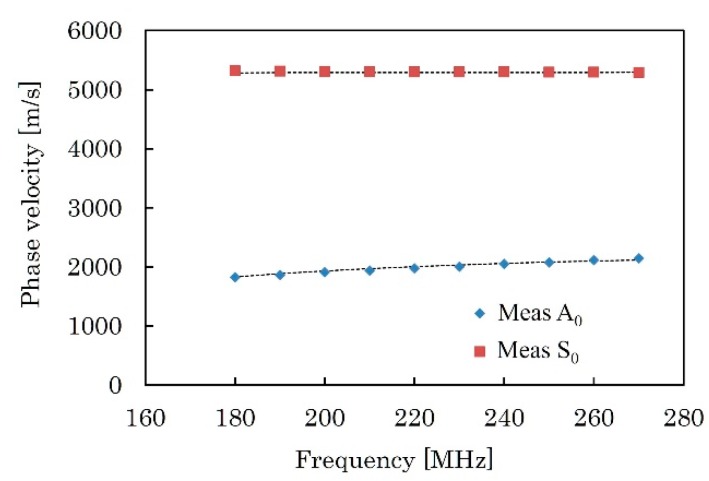
Phase velocities of A_0_ and S_0_ mode relative to measurement frequency *f* and the KNN-CT thickness *H*. Black broken lines are calculated phase velocities with best fitted parameters, as shown in Table 2.

Figure 12a shows a typical *V*(*z*) curve measured for the KNN-CT film at 225 MHz. By applying FFT to the *V*(*z*) curve, Δ*z* of 9.80 μm and 79.71 μm are obtained for A_0_ and S_0_ modes, respectively. From Equation (10), the phase velocities of 1988.91 m/s and 5225.88 m/s are calculated for A_0_ and S_0_ modes, respectively. Figure 12b shows the repeatability of the measured phase velocity at a single point. The frequency of the transducer is 225 MHz. The average values of phase velocities are 1988.46 m/s with ±2*σ* of ±0.71 m/s (±0.036%) and 5226.64 m/s with ±2*σ* of ±1.92 m/s (±0.037%) for A_0_ and S_0_ modes, respectively. *σ* is the standard deviation. Phase velocity was measured 30 times and was found to be almost stable. A similar process was repeated at different frequencies from 180 MHz to 270 MHz, where clear *V*(*z*) curve was obtained. Figure 13 shows the measured phase velocities of A_0_ and S_0_ modes at this frequency range.

### 4.3. Determination of Material Constants

**Figure 14:**
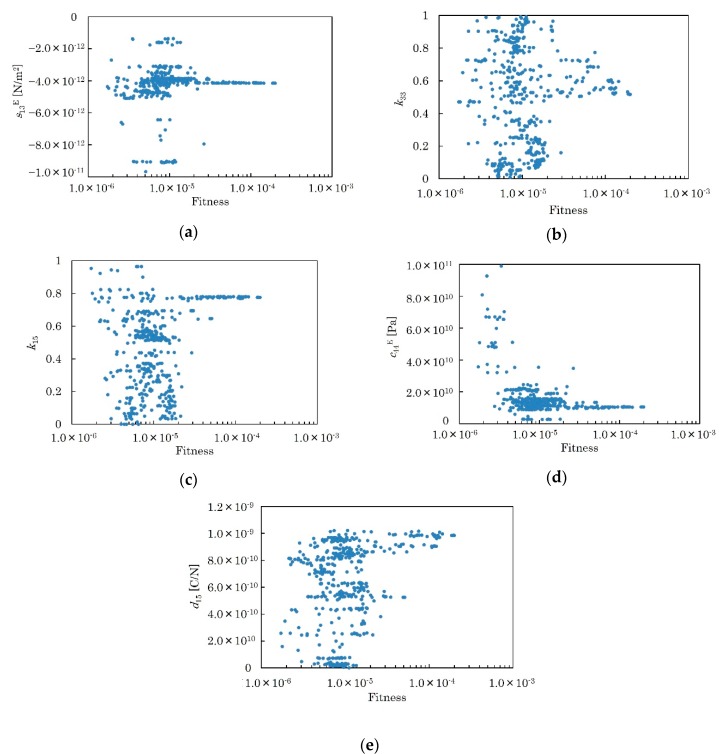
Plot of all generated fitting parameters related to fitness. (**a**) *s*_13_^E^. (**b**) *k*_33_. (**c**) *k*_15_. (**d**) *c*_44_^E^. (**e**) *d*_15_.

*k*_33_, *k*_15_, *s*_13_^E^, *c*_44_^E^ and *d*_15_ were determined by the algorithm described in Section 2.4 using the measured phase velocities shown in Figure 13. The initial values were set as *k*_33_ = 0.61, *k*_15_ = 0.64, *s*_13_^E^ = −3.43 pm^2^/N, *c*_44_^E^ = 37.0 GPa and *d*_15_ = 306 pC/N. These values are for hot-pressed pure KNN materials reported in [13]. *M* was set to 10, and the tournament size was set to 5. *P*_c_ and *P*_m_ were set to 90% and 5%, respectively. The calculation was repeated three times at different numbers of generation, and all of them were terminated after calculating all generations. The total number of generations were 500. The relationship between the fitness and the distribution of the fitting parameters in all generations were summarized in Figure 14a–e. There are many possible combinations of fitting parameters in the range of fitness less than 2 × 10^−5^. As shown in Figure 14a–e, possible combinations decreased as the fitness increased. The fitting parameters seem to converge over the fitness range of 10^−4^. The best fitness was obtained as 1.99 × 10^−4^ when *k*_33_ = 0.516, *k*_15_ = 0.776, *s*_13_^E^ = −4.15 pm^2^/N, *c*_44_^E^ = 10.5 GPa and *d*_15_ = 985 pC/N. Table 2 summarizes all the material constants of the KNN-CT film calculated by the fitting parameters, compared to [13]. By doping CT, piezoelectric and dielectric properties are enhanced, compared to the hot-pressed pure KNN. These properties are also higher than the reported ScAlN film [15]. The calculated phase velocities with the calculated material constants are in good agreement with the measured ones, as shown in Figure 13. The average of the root mean squared error of all the phase velocities over the measured frequencies $\sqrt{\Delta V^{2}}$ was 16.0 m/s at the best fitness.

## 5. Discussion

We determined all elastic constants, piezoelectric constants, and dielectric constants of the KNN-CT film. In this section, the accuracy of the determined constants is discussed.

First, a clear resonance response is needed to achieve reasonable accuracy in the measurement of resonance frequency. If the quality of a piezoelectric film is poor, the exact resonance frequencies are often difficult to determine, and the material constants become inaccurate. This is the case with the KNN-CT film prepared in this study. The thickness longitudinal mode, especially, showed a very small resonance response. As a result, the measured *c*_33_^E^ and coupling factors are significantly smaller than those of hot-pressed pure KNN, single crystal and KNN-based ceramics [13,14,27].

The second major source of inaccuracy is the measurement error of phase velocities by LFB-UFC. The measured *V*(*z*) curve of leaky Lamb wave, especially A_0_ mode, (Figure 12a), had smaller interference peaks than that of bulk materials [16,17]. This is probably because deliquescence occurs on the KNN-CT membrane during measurement by the water coupler, which fills a gap on the cylindrical lens. It is well known that alkaline niobate-based ceramics, such as KNN, shows deliquescence and a reaction with moisture in air [28,29,30].

**Figure 15:**
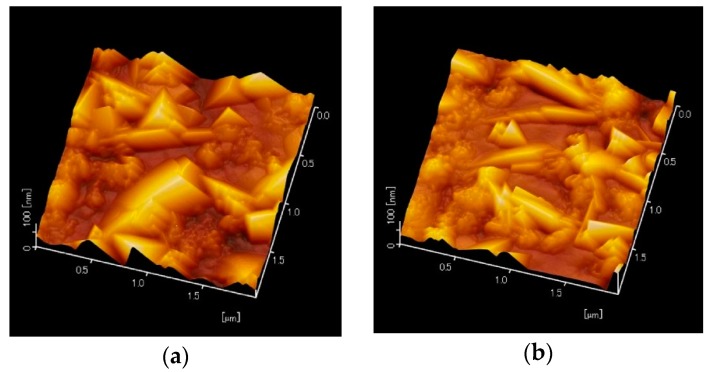
Different surface morphology of two test samples. (**a**) as-coated KNN-CT film. (**b**) after dipping into DI water for 36 h.

**Figure 16:**
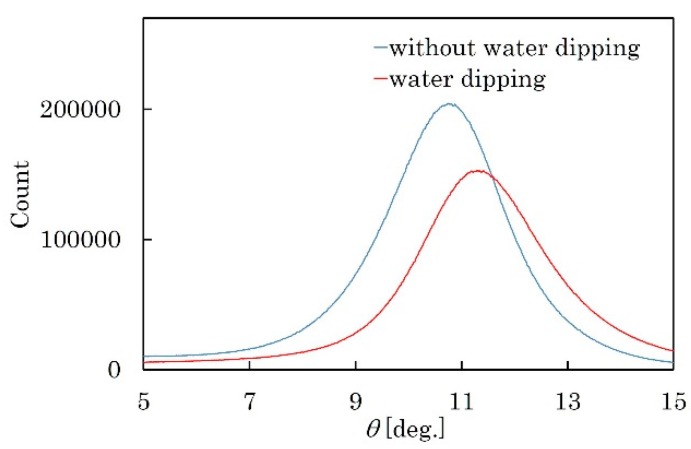
Deterioration of crystal orientation of KNN-CT film by dipping DI water. Deliquescence occurred at KNN-CT film.

To confirm the deliquescence, two of the KNN-CT-coated chips were prepared. One of them was immersed in DI water for 36 h. Surface morphology and crystal orientation were then observed by Atomic Force Microscopy (AFM) and XRD, respectively. Figure 15a,b show the surface morphology of the as-coated and water-dipped films, respectively. The tent-like crystalline structures in Figure 15b were thinner than that in Figure 15a. In Figure 16, the FWHM of the water-immersed chip and another one is 2.699° and 2.365°, respectively, which clearly shows the deterioration of crystal orientation by DI water. The KNN-CT film, therefore, has deliquescence, and thus easily reacts with moisture in the air. Although the KNN-CT membrane was covered with the Al film, the membrane was exposed to the water coupler for a long time during the LFB-UMC measurement. The crystalline deterioration may have occurred by moisture during the measurement, which decreased the interference peak. Our proposed method, therefore, may not be suitable for materials that react with water.

The third major source of inaccuracy is in the fabrication of the self-suspended Al-metalized KNN-CT membrane for the LFB-UMC measurement. The ion milling process to remove SiO_2_/Ti/Pt layers does not have a selectivity to the KNN-CT film. Therefore, the thickness of the KNN-CT film could be thinner than the original one (1.75 μm), which affects the calculation results. Moreover, the self-suspended KNN-CT membrane is easy to break during the measurement. We also prepared the self-suspended KNN-CT membrane without an Al metallization layer, but it broke during the measuring process, probably because of the deliquescence and stress caused by the water coupler. The KNN-CT membrane with the Al metallization layer survived without cracks, but we needed to consider the Al metallization layer in the calculation described in Section 2.3. The error of the material constants of Al also affects the material constants. One solution is to measure leaky Rayleigh wave instead of leaky Lamb wave, because it is not necessary to fabricate the self-suspended membrane. However, numerical calculation is more complicated, because it requires several layers (seed, adhesion, isolation and substrate). The inaccuracy of the material constants of such layers is also a source of error.

As shown in Figure 14a–e, increasing fitness is an index of the convergence of the fitting parameters. Fitness is theoretically infinite when the calculated phase velocities fit the measured sample completely. After the calculation of 500 generations, fitness was actually limited to the 10^−4^ range, where $\sqrt{\Delta V^{2}}$ is less than 20 m/s. This may be because the variance of *k*_33_ and *d*_15_ is relatively large compared to that of *s*_13_^E^, *k*_15_ and *c*_44_^E^, which are almost convergent over the fitness range of 10^−4^. Further convergence of *k*_33_ and *d*_15_ may improves the fitness.

## 6. Conclusions

In this study, we investigated the full set of material constants of a blanket KNN-CT film for the first time. First, some of the material constants were measured from the resonance responses of piezoelectric resonators, which were fabricated by the MEMS process. Next, the phase velocities of leaky Lamb wave (A_0_ and S_0_ modes) were measured on a self-suspended Al-metalized KNN-CT membrane using a LFB-UMC system. The other constants were determined by fitting the measured phase velocities with theoretical ones, which were calculated from the material constants including multiple fitting parameters, i.e., unknown constants. After the fitting using genetic algorithm, the theoretical phase velocities were well fitted with error $\sqrt{\Delta V^{2}}$ of 16.0 m/s. The KNN-CT film measured in this study was not of high quality, and the resonance response were small and unclear, especially for the thickness longitudinal mode. In addition, the KNN-CT film has deliquescence, which affected LFB-UMC measurement with a water coupler. The sample of the LFB-UMC measurement, the suspended Al-metalized KNN-CT membrane, also involves unconfident properties such as thickness and material constant of electrodes. Some of the determined material constants, especially *d*_15_, may be affected by such problems. Nevertheless, all material constants were successfully determined, and the viability of the proposed method was demonstrated.

## Figures and Tables

**Table 1:** Boundary conditions.

Layers	Boundary Conditions	Comment
Layer I and II (*x*_3_ = *H*)	*T*_31_^I^ = *T*_31_^II^ = 0	Air
	*T*_33_^I^ = *T*_33_^II^ = 0	Air
	*φ*^I^ = *φ*^II^	
	*D*_3_^I^ = *D*_3_^II^	
Layer II and III (*x*_3_ = 0)	*u*_1_^II^ = *u*_1_^III^	
	*u*_3_^II^ = *u*_3_^III^	
	*T*_31_^II^ = *T*_31_^III^	
	*T*_33_^II^ = *T*_33_^III^	
	*φ*^II^ = *φ*^III^ =0	Metalize
	*D*_3_^II^ = *D*_3_^III^	
Layer III and IV (*x*_3_ = −*h*_Al_)	*u*_3_^III^ = *u*_3_^IV^	
	*T*_31_^III^ = *T*_31_^IV^	
	*T*_33_^III^ = *T*_33_^IV^	

**Table 2:** Determined material constants of KNN-CT film, compared to a hot-pressed pure KNN [13].

	This Study	Ref. [13]	Unit
**Elastic compliance constants: *s_ij_*^E^**			
*s*_11_^E^	9.25	8.20	pN/m^2^
*s*_12_^E^	−3.05	−2.54	pN/m^2^
*s*_13_^E^	−4.15	−3.43	pN/m^2^
*s*_33_^E^	16.7	10.10	pN/m^2^
*s*_44_^E^	95.2	27.00	pN/m^2^
*s*_66_^E^	24.6	21.48	pN/m^2^
**Elastic stiffness constants: *c_ij_*^E^**			
*c*_11_^E^	162	196.7	GPa
*c*_12_^E^	80.3	103.6	GPa
*c*_13_^E^	60.1	102.0	GPa
*c*_33_^E^	89.8	168.0	GPa
*c*_44_^E^	10.5	37.0	GPa
*c*_66_^E^	40.6	46.6	GPa
**Piezoelectric constants: *e_ij_*, *d_ij_***			
*e*_15_	10.3	11.30	C/m^2^
*e*_31_	−5.67	−2.36	C/m^2^
*e*_33_	10.6	9.80	C/m^2^
*d*_15_	985	306	pC/N
*d*_31_	−79.0	−51	pC/N
*d*_33_	223	127	pC/N
**Relative dielectric constants: *ɛ_ij_*^S^ (ɛ_0_), *ɛ_ij_*^T^ (ɛ_0_)**			
*ɛ*_11_^S^	760	545	-
*ɛ*_33_^S^	901	306	-
*ɛ*_11_^T^	1911	496	-
*ɛ*_33_^T^	1270	938	-
